# 14-Benzoyl­mesaconine hydro­chloride methanol monosolvate

**DOI:** 10.1107/S1600536811010300

**Published:** 2011-03-26

**Authors:** Yan Mu, Lin Li, Hai-Liu Wei, Tong-Chun Kuang, Song-Qing Hu

**Affiliations:** a College of Light Industry and Food Sciences, South China University of Technology, Guangzhou 510640, People’s Republic of China; b Analytical and Testing Center, South China University of Technology, Guangzhou 510640, People’s Republic of China

## Abstract

The title compound, C_31_H_44_N_3_O_10_^+^·Cl^−^·CH_4_O, is the methanol solvate of 8-benzo­yloxy-,9,11,11a-tetra­hydroxy-6,10,13-trimeth­oxy-3-meth­oxy­methyl-1-methyl­tetra­deca­hydro-1*H*-3,6a,12-(epiethane-1,1,2-tri­yl)-7,9-methanona­phtho[2,3-*b*]azocin-1-ium chloride, the amine-protonated hydro­chloride of 14-benzoyl­mesaconine hydro­chloride. The cation has an aconitine carbon skeleton with four six-membered rings of which three display chair conformations and one a boat conformation, and two five-membered rings with envelope conformations. In the crystal, the components are connected into an infinite chain by inter- and intra­molecular O—H⋯O, N—H⋯O and O—H⋯Cl hydrogen bonds.

## Related literature

For general background to diterpenoid alkaloids, see: Ameri (1998 ▶); Desai *et al.* (1998 ▶); Suzuki *et al.* (1994 ▶). For the chemical structure of the title compound established from MS data, see: Zhang *et al.* (2005 ▶); Wang *et al.* (2009 ▶); Yue *et al.* (2009 ▶). For background to the strong toxicity of *Aconitum* alkaloids, see: Zhang *et al.* (2002 ▶). For ring numbering and ring conformations of the title compound, see: He *et al.* (2008 ▶).
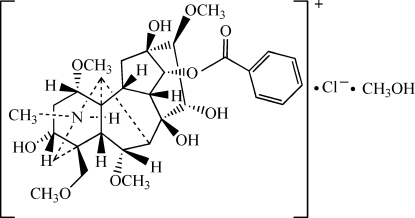


## Experimental

### 

#### Crystal data

C_31_H_44_NO_10_^+^·Cl^−^·CH_4_O
*M* *_r_* = 658.16
Orthorhombic, 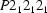

*a* = 12.919 (3) Å
*b* = 15.748 (3) Å
*c* = 16.045 (3) Å
*V* = 3264.2 (11) Å^3^
*Z* = 4
Mo *K*α radiation
μ = 0.18 mm^−1^
*T* = 123 K
0.30 × 0.20 × 0.20 mm

#### Data collection

Rigaku/MSC Mercury CCD diffractometer
Absorption correction: multi-scan (REQAB; Jacobson, 1998 ▶) *T*_min_ = 0.969, *T*_max_ = 0.977
29100 measured reflections
6908 independent reflections
4585 reflections with *I* > 2σ(*I*)
*R*_int_ = 0.050

#### Refinement

*R*[*F* ^2^ > 2σ(*F* ^2^)] = 0.047
*wR*(*F* ^2^) = 0.151
*S* = 1.10
6908 reflections
422 parameters
1 restraint
H atoms treated by a mixture of independent and constrained refinement
Δρ_max_ = 0.36 e Å^−3^
Δρ_min_ = −0.50 e Å^−3^
Absolute structure: Flack (1983 ▶), 1726 Friedel pairs
Flack parameter: −0.01 (11)

### 

Data collection: *RAPID-AUTO* (Rigaku, 1998 ▶); cell refinement: *RAPID-AUTO*; data reduction: *CrystalStructure* (Rigaku/MSC, 2002 ▶); program(s) used to solve structure: *SHELXS97* (Sheldrick, 2008 ▶); program(s) used to refine structure: *SHELXL97* (Sheldrick, 2008 ▶); molecular graphics: *ORTEPII* (Johnson, 1976 ▶); software used to prepare material for publication: *SHELXL97*.

## Supplementary Material

Crystal structure: contains datablocks I, global. DOI: 10.1107/S1600536811010300/zl2352sup1.cif

Structure factors: contains datablocks I. DOI: 10.1107/S1600536811010300/zl2352Isup2.hkl

Additional supplementary materials:  crystallographic information; 3D view; checkCIF report

## Figures and Tables

**Table 1:** Hydrogen-bond geometry (Å, °)

*D*—H⋯*A*	*D*—H	H⋯*A*	*D*⋯*A*	*D*—H⋯*A*
O3—H3*A*⋯O5	0.82	2.11	2.612 (4)	119
O3—H3*A*⋯O11	0.82	2.22	2.923 (4)	144
O4—H4*A*⋯Cl1	0.82	2.30	3.083 (3)	161
O6—H6⋯Cl1	0.82	2.40	3.197 (3)	165
O9—H9⋯O11^i^	0.82	1.98	2.776 (4)	163
N1—H1⋯O8	0.90 (2)	2.11 (4)	2.808 (4)	134 (4)
N1—H1⋯O9	0.90 (2)	2.20 (4)	2.795 (5)	123 (4)
O11—H11⋯Cl1^ii^	0.82	2.30	3.084 (3)	158

Symmetry codes: (i) 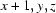; (ii) 
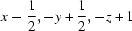
.

**Fig. 1.**
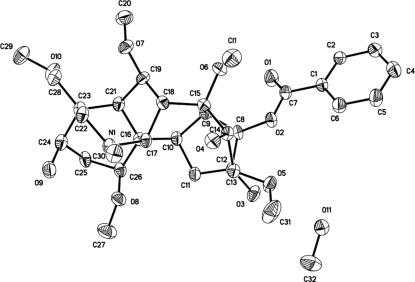
Thermal ellipsoid plot of the title compound with the atom numbering scheme. Displacement ellipsoids are drawn at the 30% probability level. All H atoms were omitted for clarity.

**Fig. 2.**
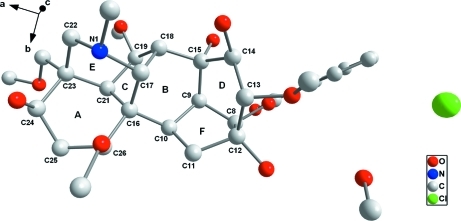
View of the structure of the title compound with naming of the rings (He et al., 2008), showing 50% probability displacement ellipsoids.

**Fig. 3.**
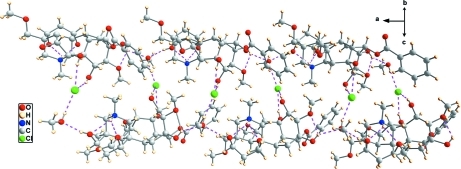
View of a one-dimensional chain of the title compound that stretches along to the a axis of the cell. O—H···O, N—H···O and O—H···Cl hydrogen bonds interactions are shown as dashed pink lines.

## supplementary crystallographic information

### Comment

*Aconitum* species such as e.g. *Aconitum Caremichaeli Debx.* have been widely used in Chinese Traditional Medicine, and its tubers and roots have found therapeutical use for the treatment of for example rheumatic pain, rheumatoid arthritis and some other inflammations. Aconitine-type alkaloid extracts from the roots of *Aconitum Caremichaeli Debx.* are reported to have, among others, analgetic, diuretics, anti-inflammatory and cardiotonic properties (Ameri, 1998; Desai *et al.*, 1998; Suzuki *et al.*, 1994). At the same time, owing to their strong toxicity, poisoning with these *Aconitum* alkaloids does frequently happen (Zhang *et al.*, 2002). Therefore, in order to better understand the pharmacology of the *Aconitum* alkaloids it is desirable to establish their molecular structures. The diterpenoid alkaloid 14-benzoylmesaconine, the title compound, was previously isolated from *Aconitum Caremichaeli Debx.*, and its structure was established from the MS data (Zhang *et al.*, 2005; Wang *et al.*, 2009; Yue *et al.*, 2009). However, there are no reports on the crystal structure or the NMR data of 14-benzoylmesaconine. In this paper, we would like to present the crystal structure and NMR data of the title compound, the hydrochloride salt of 14-benzoylmesaconine as its mono methanol solvate.

The molecular structure of the title compound is shown in Fig. 1. There is one cation, protonated at the amine N atom, one chloride anion and one methanol solvate molecule in the asymmetric unit of the structure. The bond lengths and angles are in good agreement with expected values. For ring naming (Figure 2) and ring conformations please refer to He *et al.* (2008). The cation of the title compound has an aconitine carbon skeleton with four six-membered rings and two five-membered rings. Six-membered rings A (C16/C21/C23/C24/C25/C26) and B (C9/C10/C15/C16/C17/C18) adopt chair conformations, six-membered heterocyclic ring E (N1/C17/C21/C22/C23) adopts a chair conformation, and the six-membered ring D (C8/C9/C12/C13/C14/C15) adopts a boat conformation. The two five-membered rings C (C16/C17/C18/C19/C21) and F (C8/C9/C10/C11/C12) adopt envelope conformations (Fig. 2). Intermolecular O—H···O hydrogen bonds between the hydroxyls of the cations and the hydroxy O atoms of the methanol solvate molecules lead to infinite chains. The chains are further connected with each other via intermolecular O—H···Cl hydrogen bonding interactions involving the hydroxyls of methanol molecules and the cations as donors and the chloride anions as acceptors to form 1D double-chains along to the *a* axis (Table 1, Fig. 3). Within the hydrogen-bonded double-chain the chloride anions are bonded to three hydroxyl groups, two from one cation and another one from a methanol solvate molecule. Each cation donates four hydrogen bonds to two methanol solvate molecules and one chloride anion, whilst each methanol solvate molecule donates one hydrogen bond to chloride anion and acceptes two hydrogen bonds from two hydroxyl groups of two cations. In addition, intramolecular N—H···O and O—H···O hydrogen bonds are also observed within the double-chain. Finally, the chains are linked into a three-dimensional supramolecular network through intermolecular O—H···O and O—H···Cl hydrogen-bonding interactions.

### Experimental

Air-dried and powdered roots of *Aconitum Caremichaeli Debx.* (20 kg) were extracted with 90% EtOH (40 *L*) under reflux with a Soxhlet extractor. After the removal of solvent, the extract residue (200 g) was successively eluted in a macroporous resin by elution with water (5 *L*), 30% EtOH (5 *L*), 60% EtOH (5 *L*) and 90% EtOH (5 *L*), and different extract fractions were obtained. From the 30% EtOH fraction, a black amorphous powder (50 g) was obtained after the removal of the solvent under reduced pressure, which then was chromatographed on a silica gel (200–300 mesh) column, eluted with a chloroform-MeOH (99:1 to 1:1) gradient system, to give 25 fractions (2 *L* per fraction). The title compound (2 g) can be isolated between the eighteenth and twentieth fractions (yield 0.01%). Single crystals suitable for X-ray diffraction were prepared by slow evaporation of a solution of the title compound in CHCl_3_ at room temperature. [^1^H NMR(CDCl_3_, δ, p.p.m.) 8.08 (d, 2H), 7.58 (t, 1H), 7.45 (t, 2H), 5.01 (d, 1H), 4.58 (d, 1H), 4.17 (t, 2H), 3.70 (s, 3H), 3.57 (d, 2H), 3.39 (t, 2H), 3.30–3.34 (m, 15H), 3.21 (d, 1H), 2.94 (s, 3H), 2.56 (s, 2H), 2.40 (d, 2H), 2.23 (t, 2H), 1.81 (d, 1H), 1.56 (d, 1H); ^13^C NMR (CDCl_3_, δ, p.p.m.) 167.88, 134.45, 131.68, 131.20, 129.66, 93.60, 83.20, 81.48, 83.20, 81.48, 80.90, 79.15, 78.38, 76.36, 70.79, 68.38, 62.44, 59.67, 58.76, 55.76, 53.13, 52.06, 50.07, 45.55, 44.86, 43.76, 42.20, 41.91, 37.41, 30.62].

### Refinement

The hydrogen atoms of the NH group were refined using bond distance restraints (N—H = 0.87 (2) Å). All other H atoms were located in difference density maps, and were treated as riding atoms with C—H and O—H distances of 0.93, 0.96, 0.97, 0.98 and 0.82 Å, for aryl, methyl, methine, CH and OH, respectively, with *U*_iso_(H) = 1.5*U*_eq_(C) for methyl and 1.5*U*_eq_(O) for hydroxy, and 1.2*U*_eq_(C) for the others. Methyl and hydroxyl hydrogen atoms were allowed to rotate at a fixed angle around the C—O bond to best fit the experimental electron density.

### Crystal data

**Table:**

C_31_H_44_NO_10_^+^·Cl^−^·CH_4_O	*F*(000) = 1408
*M**_r_* = 658.16	*D*_x_ = 1.339 Mg m^−^^3^
Orthorhombic, *P*2_1_2_1_2_1_	Mo *K*α radiation, λ = 0.71073 Å
Hall symbol: P 2ac 2ab	Cell parameters from 5837 reflections
*a* = 12.919 (3) Å	θ = 2.8–27.9°
*b* = 15.748 (3) Å	µ = 0.18 mm^−^^1^
*c* = 16.045 (3) Å	*T* = 123 K
*V* = 3264.2 (11) Å^3^	Block, colorless
*Z* = 4	0.30 × 0.20 × 0.20 mm

### Data collection

**Table:**

Rigaku/MSC Mercury CCD diffractometer	6908 independent reflections
Radiation source: fine-focus sealed tube	4585 reflections with *I* > 2σ(*I*)
graphite	*R*_int_ = 0.050
ω scans	θ_max_ = 26.8°, θ_min_ = 3.0°
Absorption correction: multi-scan (REQAB; Jacobson, 1998)	*h* = −16→16
*T*_min_ = 0.969, *T*_max_ = 0.977	*k* = −19→19
29100 measured reflections	*l* = −20→20

### Refinement

**Table:**

Refinement on *F*^2^	Hydrogen site location: inferred from neighbouring sites
Least-squares matrix: full	H atoms treated by a mixture of independent and constrained refinement
*R*[*F*^2^ > 2σ(*F*^2^)] = 0.047	*w* = 1/[σ^2^(*F*_o_^2^) + (0.040*P*)^2^ + 3.5*P*] where *P* = (*F*_o_^2^ + 2*F*_c_^2^)/3
*wR*(*F*^2^) = 0.151	(Δ/σ)_max_ = 0.001
*S* = 1.10	Δρ_max_ = 0.36 e Å^−^^3^
6908 reflections	Δρ_min_ = −0.50 e Å^−^^3^
422 parameters	Extinction correction: *SHELXL97* (Sheldrick, 2008), Fc^*^=kFc[1+0.001xFc^2^λ^3^/sin(2θ)]^-1/4^
1 restraint	Extinction coefficient: 0.0187 (13)
Primary atom site location: structure-invariant direct methods	Absolute structure: Flack (1983), 1726 Friedel pairs
Secondary atom site location: difference Fourier map	Flack parameter: −0.01 (11)

### Special details

**Table:**

Geometry. All e.s.d.'s (except the e.s.d. in the dihedral angle between two l.s. planes) are estimated using the full covariance matrix. The cell e.s.d.'s are taken into account individually in the estimation of e.s.d.'s in distances, angles and torsion angles; correlations between e.s.d.'s in cell parameters are only used when they are defined by crystal symmetry. An approximate (isotropic) treatment of cell e.s.d.'s is used for estimating e.s.d.'s involving l.s. planes.
Refinement. Refinement of *F*^2^ against ALL reflections. The weighted *R*-factor *wR* and goodness of fit *S* are based on *F*^2^, conventional *R*-factors *R* are based on *F*, with *F* set to zero for negative *F*^2^. The threshold expression of *F*^2^ > σ(*F*^2^) is used only for calculating *R*-factors(gt) *etc*. and is not relevant to the choice of reflections for refinement. *R*-factors based on *F*^2^ are statistically about twice as large as those based on *F*, and *R*- factors based on ALL data will be even larger.

### Fractional atomic coordinates and isotropic or equivalent isotropic displacement parameters (Å^2^)

**Table:**

	*x*	*y*	*z*	*U*_iso_*/*U*_eq_	
O1	0.2155 (3)	0.3580 (2)	0.1441 (2)	0.0676 (9)	
O2	0.2226 (2)	0.37571 (18)	0.28249 (16)	0.0457 (7)	
O3	0.2193 (2)	0.53973 (19)	0.3747 (2)	0.0562 (8)	
H3A	0.1791	0.5138	0.4050	0.084*	
O4	0.4338 (2)	0.30793 (17)	0.47185 (17)	0.0462 (7)	
H4A	0.4359	0.2560	0.4693	0.069*	
O5	0.2329 (2)	0.40600 (19)	0.47032 (18)	0.0525 (7)	
O6	0.4216 (2)	0.26331 (16)	0.27517 (17)	0.0448 (7)	
H6	0.4352	0.2220	0.3044	0.067*	
O7	0.6872 (2)	0.28611 (17)	0.23706 (19)	0.0500 (7)	
O8	0.6159 (2)	0.59029 (17)	0.42349 (19)	0.0523 (7)	
O9	0.8284 (2)	0.5542 (2)	0.4047 (2)	0.0591 (8)	
H9	0.8901	0.5451	0.4126	0.089*	
O10	0.8654 (2)	0.4637 (2)	0.1640 (2)	0.0633 (9)	
N1	0.6874 (2)	0.4220 (2)	0.4292 (2)	0.0434 (8)	
H1	0.696 (4)	0.4762 (16)	0.446 (3)	0.076 (17)*	
C1	0.0994 (3)	0.2805 (2)	0.2312 (2)	0.0414 (9)	
C2	0.0520 (3)	0.2385 (3)	0.1659 (3)	0.0452 (9)	
H2	0.0758	0.2469	0.1118	0.054*	
C3	−0.0302 (3)	0.1842 (3)	0.1797 (3)	0.0560 (11)	
H3	−0.0619	0.1566	0.1353	0.067*	
C4	−0.0649 (4)	0.1712 (3)	0.2602 (3)	0.0586 (11)	
H4	−0.1205	0.1349	0.2699	0.070*	
C5	−0.0174 (4)	0.2119 (3)	0.3265 (3)	0.0595 (12)	
H5	−0.0404	0.2027	0.3807	0.071*	
C6	0.0649 (3)	0.2668 (3)	0.3116 (3)	0.0517 (10)	
H6A	0.0968	0.2943	0.3559	0.062*	
C7	0.1852 (3)	0.3401 (3)	0.2127 (3)	0.0450 (9)	
C8	0.2993 (3)	0.4412 (3)	0.2759 (2)	0.0410 (9)	
H8	0.2795	0.4810	0.2317	0.049*	
C9	0.4112 (3)	0.4122 (2)	0.2622 (2)	0.0394 (8)	
H9A	0.4209	0.3946	0.2042	0.047*	
C10	0.4718 (3)	0.4964 (2)	0.2794 (3)	0.0410 (9)	
H10	0.4736	0.5284	0.2270	0.049*	
C11	0.3996 (3)	0.5450 (2)	0.3401 (3)	0.0468 (10)	
H11A	0.4360	0.5578	0.3915	0.056*	
H11B	0.3771	0.5980	0.3152	0.056*	
C12	0.3068 (3)	0.4881 (2)	0.3577 (3)	0.0423 (9)	
C13	0.3285 (3)	0.4250 (2)	0.4295 (3)	0.0424 (9)	
H13	0.3749	0.4523	0.4697	0.051*	
C14	0.3773 (3)	0.3403 (2)	0.4020 (2)	0.0380 (8)	
H14	0.3197	0.3009	0.3927	0.046*	
C15	0.4424 (3)	0.3400 (2)	0.3210 (2)	0.0380 (8)	
C16	0.5854 (3)	0.4909 (2)	0.3125 (2)	0.0403 (9)	
C17	0.5845 (3)	0.4256 (2)	0.3837 (2)	0.0388 (9)	
H17	0.5283	0.4382	0.4229	0.047*	
C18	0.5609 (3)	0.3453 (2)	0.3357 (2)	0.0379 (8)	
H18A	0.5859	0.2952	0.3658	0.045*	
C19	0.6176 (3)	0.3550 (2)	0.2510 (3)	0.0411 (9)	
H19	0.5653	0.3538	0.2067	0.049*	
C20	0.7003 (4)	0.2693 (3)	0.1503 (3)	0.0655 (13)	
H20A	0.6348	0.2541	0.1263	0.098*	
H20B	0.7483	0.2234	0.1431	0.098*	
H20C	0.7265	0.3192	0.1231	0.098*	
C21	0.6640 (3)	0.4455 (2)	0.2540 (3)	0.0413 (9)	
H21	0.6640	0.4715	0.1985	0.050*	
C22	0.7736 (3)	0.3949 (3)	0.3727 (3)	0.0470 (10)	
H22A	0.7645	0.3358	0.3574	0.056*	
H22B	0.8393	0.4004	0.4015	0.056*	
C23	0.7741 (3)	0.4501 (3)	0.2944 (3)	0.0449 (9)	
C24	0.8054 (3)	0.5448 (3)	0.3170 (3)	0.0510 (10)	
H24	0.8672	0.5601	0.2850	0.061*	
C25	0.7206 (3)	0.6064 (3)	0.2961 (3)	0.0488 (10)	
H25A	0.7130	0.6090	0.2360	0.059*	
H25B	0.7405	0.6625	0.3153	0.059*	
C26	0.6178 (3)	0.5833 (2)	0.3343 (3)	0.0450 (9)	
H26	0.5653	0.6218	0.3117	0.054*	
C27	0.6260 (5)	0.6758 (3)	0.4529 (4)	0.0805 (17)	
H27A	0.6957	0.6950	0.4442	0.121*	
H27B	0.6101	0.6778	0.5113	0.121*	
H27C	0.5791	0.7118	0.4230	0.121*	
C28	0.8615 (3)	0.4165 (3)	0.2395 (3)	0.0529 (11)	
H28A	0.9270	0.4215	0.2688	0.064*	
H28B	0.8499	0.3569	0.2272	0.064*	
C29	0.9648 (4)	0.4617 (4)	0.1275 (3)	0.0717 (15)	
H29A	1.0140	0.4871	0.1648	0.108*	
H29B	0.9638	0.4927	0.0760	0.108*	
H29C	0.9842	0.4038	0.1168	0.108*	
C30	0.6832 (4)	0.3678 (3)	0.5058 (3)	0.0565 (11)	
H30A	0.6665	0.3105	0.4904	0.085*	
H30B	0.6311	0.3893	0.5429	0.085*	
H30C	0.7492	0.3688	0.5332	0.085*	
C31	0.2372 (4)	0.4038 (5)	0.5574 (3)	0.093 (2)	
H31A	0.2842	0.3600	0.5748	0.140*	
H31B	0.1695	0.3922	0.5793	0.140*	
H31C	0.2609	0.4576	0.5780	0.140*	
Cl1	0.44750 (9)	0.12099 (7)	0.41575 (8)	0.0623 (3)	
O11	0.0250 (2)	0.51954 (19)	0.46583 (18)	0.0553 (8)	
H11	0.0125	0.4738	0.4882	0.083*	
C32	0.0388 (5)	0.5825 (4)	0.5277 (3)	0.0859 (18)	
H32A	0.1112	0.5888	0.5394	0.129*	
H32B	0.0030	0.5659	0.5776	0.129*	
H32C	0.0115	0.6355	0.5081	0.129*	

### Atomic displacement parameters (Å^2^)

**Table:**

	*U*^11^	*U*^22^	*U*^33^	*U*^12^	*U*^13^	*U*^23^
O1	0.065 (2)	0.091 (3)	0.0468 (18)	−0.0232 (19)	0.0066 (16)	0.0031 (17)
O2	0.0363 (14)	0.0545 (16)	0.0463 (16)	−0.0098 (13)	−0.0066 (12)	0.0044 (13)
O3	0.0364 (15)	0.0546 (18)	0.078 (2)	0.0108 (14)	0.0071 (15)	0.0074 (15)
O4	0.0481 (16)	0.0467 (15)	0.0437 (16)	0.0047 (13)	−0.0087 (13)	0.0078 (12)
O5	0.0400 (15)	0.0694 (19)	0.0480 (17)	−0.0030 (14)	0.0094 (13)	0.0029 (14)
O6	0.0535 (16)	0.0353 (13)	0.0455 (16)	−0.0068 (13)	−0.0035 (13)	−0.0059 (12)
O7	0.0474 (15)	0.0444 (16)	0.0583 (19)	0.0102 (13)	0.0082 (14)	0.0005 (13)
O8	0.0550 (17)	0.0406 (14)	0.0613 (19)	−0.0047 (13)	−0.0044 (15)	−0.0061 (13)
O9	0.0442 (16)	0.0607 (19)	0.073 (2)	−0.0027 (15)	−0.0088 (15)	−0.0015 (16)
O10	0.0484 (17)	0.078 (2)	0.064 (2)	0.0129 (16)	0.0109 (15)	0.0216 (17)
N1	0.0363 (17)	0.047 (2)	0.047 (2)	0.0004 (15)	−0.0043 (14)	0.0067 (16)
C1	0.0357 (19)	0.044 (2)	0.044 (2)	−0.0035 (17)	−0.0020 (17)	−0.0013 (17)
C2	0.038 (2)	0.049 (2)	0.049 (2)	0.0003 (18)	−0.0015 (18)	−0.0070 (18)
C3	0.049 (2)	0.056 (3)	0.063 (3)	−0.003 (2)	−0.002 (2)	−0.010 (2)
C4	0.051 (3)	0.055 (3)	0.069 (3)	−0.008 (2)	−0.009 (2)	0.000 (2)
C5	0.051 (2)	0.070 (3)	0.058 (3)	−0.015 (2)	0.005 (2)	0.008 (2)
C6	0.049 (2)	0.063 (3)	0.042 (2)	−0.009 (2)	−0.001 (2)	0.001 (2)
C7	0.0338 (19)	0.052 (2)	0.049 (2)	−0.0007 (18)	−0.0068 (18)	−0.0001 (19)
C8	0.0301 (18)	0.044 (2)	0.049 (2)	−0.0122 (16)	−0.0049 (16)	0.0044 (18)
C9	0.0366 (19)	0.040 (2)	0.042 (2)	−0.0006 (16)	−0.0022 (16)	0.0031 (16)
C10	0.0326 (19)	0.0377 (19)	0.053 (2)	−0.0016 (16)	−0.0046 (17)	0.0052 (17)
C11	0.034 (2)	0.039 (2)	0.067 (3)	−0.0009 (17)	−0.0008 (18)	−0.0007 (19)
C12	0.0273 (17)	0.043 (2)	0.056 (2)	0.0056 (16)	0.0023 (17)	0.0014 (18)
C13	0.0355 (19)	0.045 (2)	0.046 (2)	−0.0002 (17)	0.0008 (17)	0.0011 (17)
C14	0.0357 (18)	0.044 (2)	0.0344 (19)	−0.0005 (17)	−0.0001 (16)	0.0054 (16)
C15	0.0406 (19)	0.0355 (18)	0.038 (2)	−0.0005 (17)	−0.0038 (16)	−0.0002 (15)
C16	0.040 (2)	0.0340 (19)	0.047 (2)	0.0036 (16)	−0.0041 (17)	0.0042 (16)
C17	0.0305 (18)	0.040 (2)	0.046 (2)	0.0014 (16)	−0.0049 (16)	0.0057 (16)
C18	0.0368 (19)	0.0338 (17)	0.043 (2)	0.0000 (16)	−0.0075 (17)	0.0036 (15)
C19	0.039 (2)	0.038 (2)	0.046 (2)	0.0066 (16)	0.0077 (17)	0.0015 (16)
C20	0.069 (3)	0.065 (3)	0.063 (3)	0.004 (3)	0.011 (3)	−0.010 (2)
C21	0.036 (2)	0.039 (2)	0.049 (2)	−0.0008 (16)	−0.0003 (17)	0.0087 (17)
C22	0.0331 (19)	0.052 (2)	0.056 (3)	0.0042 (18)	−0.0018 (18)	0.0094 (19)
C23	0.038 (2)	0.043 (2)	0.054 (2)	−0.0018 (17)	−0.0015 (18)	0.0083 (18)
C24	0.037 (2)	0.052 (2)	0.064 (3)	0.0022 (19)	−0.002 (2)	0.008 (2)
C25	0.038 (2)	0.042 (2)	0.066 (3)	−0.0072 (18)	−0.0068 (19)	0.0109 (19)
C26	0.039 (2)	0.041 (2)	0.055 (3)	−0.0004 (17)	−0.0077 (18)	0.0049 (18)
C27	0.102 (4)	0.052 (3)	0.087 (4)	−0.016 (3)	0.001 (3)	−0.017 (3)
C28	0.040 (2)	0.055 (3)	0.063 (3)	0.006 (2)	0.008 (2)	0.009 (2)
C29	0.060 (3)	0.080 (3)	0.075 (3)	0.007 (3)	0.028 (3)	0.020 (3)
C30	0.057 (3)	0.066 (3)	0.047 (2)	0.003 (2)	−0.005 (2)	0.014 (2)
C31	0.062 (3)	0.171 (7)	0.047 (3)	0.008 (4)	0.011 (2)	0.017 (4)
Cl1	0.0678 (7)	0.0509 (6)	0.0683 (8)	0.0090 (6)	0.0048 (6)	0.0101 (5)
O11	0.0571 (18)	0.0564 (18)	0.0523 (18)	−0.0034 (16)	−0.0013 (15)	0.0072 (14)
C32	0.128 (5)	0.073 (3)	0.057 (3)	−0.012 (4)	−0.008 (3)	−0.008 (3)

### Geometric parameters (Å, °)

**Table:**

O1—C7	1.202 (5)	C13—H13	0.9800
O2—C7	1.342 (5)	C14—C15	1.548 (5)
O2—C8	1.434 (4)	C14—H14	0.9800
O3—C12	1.420 (4)	C15—C18	1.552 (5)
O3—H3A	0.8200	C16—C17	1.537 (5)
O4—C14	1.431 (4)	C16—C26	1.553 (5)
O4—H4A	0.8200	C16—C21	1.557 (6)
O5—C31	1.399 (5)	C17—C18	1.512 (5)
O5—C13	1.430 (4)	C17—H17	0.9800
O6—C15	1.439 (4)	C18—C19	1.551 (5)
O6—H6	0.8200	C18—H18A	0.9800
O7—C19	1.426 (4)	C19—C21	1.548 (5)
O7—C20	1.427 (5)	C19—H19	0.9800
O8—C27	1.433 (5)	C20—H20A	0.9600
O8—C26	1.436 (5)	C20—H20B	0.9600
O9—C24	1.445 (5)	C20—H20C	0.9600
O9—H9	0.8200	C21—C23	1.565 (5)
O10—C29	1.412 (5)	C21—H21	0.9800
O10—C28	1.423 (5)	C22—C23	1.528 (5)
N1—C22	1.498 (5)	C22—H22A	0.9700
N1—C30	1.497 (5)	C22—H22B	0.9700
N1—C17	1.518 (5)	C23—C28	1.526 (6)
N1—H1	0.901 (19)	C23—C24	1.587 (6)
C1—C2	1.382 (5)	C24—C25	1.501 (5)
C1—C6	1.383 (6)	C24—H24	0.9800
C1—C7	1.482 (5)	C25—C26	1.507 (5)
C2—C3	1.381 (6)	C25—H25A	0.9700
C2—H2	0.9300	C25—H25B	0.9700
C3—C4	1.383 (7)	C26—H26	0.9800
C3—H3	0.9300	C27—H27A	0.9600
C4—C5	1.386 (6)	C27—H27B	0.9600
C4—H4	0.9300	C27—H27C	0.9600
C5—C6	1.390 (6)	C28—H28A	0.9700
C5—H5	0.9300	C28—H28B	0.9700
C6—H6A	0.9300	C29—H29A	0.9600
C8—C12	1.509 (6)	C29—H29B	0.9600
C8—C9	1.532 (5)	C29—H29C	0.9600
C8—H8	0.9800	C30—H30A	0.9600
C9—C15	1.531 (5)	C30—H30B	0.9600
C9—C10	1.564 (5)	C30—H30C	0.9600
C9—H9A	0.9800	C31—H31A	0.9600
C10—C11	1.552 (6)	C31—H31B	0.9600
C10—C16	1.563 (5)	C31—H31C	0.9600
C10—H10	0.9800	O11—C32	1.414 (6)
C11—C12	1.523 (5)	O11—H11	0.8200
C11—H11A	0.9700	C32—H32A	0.9600
C11—H11B	0.9700	C32—H32B	0.9600
C12—C13	1.547 (5)	C32—H32C	0.9600
C13—C14	1.540 (5)		
			
C7—O2—C8	119.2 (3)	N1—C17—H17	110.3
C12—O3—H3A	109.5	C16—C17—H17	110.3
C14—O4—H4A	109.5	C17—C18—C19	105.6 (3)
C31—O5—C13	115.3 (4)	C17—C18—C15	108.7 (3)
C15—O6—H6	109.5	C19—C18—C15	109.8 (3)
C19—O7—C20	111.6 (3)	C17—C18—H18A	110.9
C27—O8—C26	113.5 (3)	C19—C18—H18A	110.9
C24—O9—H9	109.5	C15—C18—H18A	110.9
C29—O10—C28	111.9 (3)	O7—C19—C21	117.5 (3)
C22—N1—C30	111.2 (3)	O7—C19—C18	111.1 (3)
C22—N1—C17	111.8 (3)	C21—C19—C18	104.2 (3)
C30—N1—C17	112.6 (3)	O7—C19—H19	107.9
C22—N1—H1	111 (3)	C21—C19—H19	107.9
C30—N1—H1	108 (3)	C18—C19—H19	107.9
C17—N1—H1	103 (3)	O7—C20—H20A	109.5
C2—C1—C6	119.3 (4)	O7—C20—H20B	109.5
C2—C1—C7	118.9 (4)	H20A—C20—H20B	109.5
C6—C1—C7	121.8 (4)	O7—C20—H20C	109.5
C1—C2—C3	121.0 (4)	H20A—C20—H20C	109.5
C1—C2—H2	119.5	H20B—C20—H20C	109.5
C3—C2—H2	119.5	C19—C21—C16	100.9 (3)
C2—C3—C4	119.4 (4)	C19—C21—C23	114.0 (3)
C2—C3—H3	120.3	C16—C21—C23	108.8 (3)
C4—C3—H3	120.3	C19—C21—H21	110.9
C3—C4—C5	120.3 (4)	C16—C21—H21	110.9
C3—C4—H4	119.9	C23—C21—H21	110.9
C5—C4—H4	119.9	N1—C22—C23	109.8 (3)
C4—C5—C6	119.6 (4)	N1—C22—H22A	109.7
C4—C5—H5	120.2	C23—C22—H22A	109.7
C6—C5—H5	120.2	N1—C22—H22B	109.7
C1—C6—C5	120.3 (4)	C23—C22—H22B	109.7
C1—C6—H6A	119.8	H22A—C22—H22B	108.2
C5—C6—H6A	119.8	C28—C23—C22	106.3 (3)
O1—C7—O2	123.3 (4)	C28—C23—C21	114.7 (3)
O1—C7—C1	125.1 (4)	C22—C23—C21	108.1 (3)
O2—C7—C1	111.5 (3)	C28—C23—C24	105.6 (3)
O2—C8—C12	109.4 (3)	C22—C23—C24	110.3 (3)
O2—C8—C9	116.6 (3)	C21—C23—C24	111.7 (3)
C12—C8—C9	102.1 (3)	O9—C24—C25	107.5 (4)
O2—C8—H8	109.5	O9—C24—C23	111.8 (3)
C12—C8—H8	109.5	C25—C24—C23	111.7 (3)
C9—C8—H8	109.5	O9—C24—H24	108.6
C8—C9—C15	112.4 (3)	C25—C24—H24	108.6
C8—C9—C10	101.2 (3)	C23—C24—H24	108.6
C15—C9—C10	112.9 (3)	C24—C25—C26	113.3 (3)
C8—C9—H9A	110.0	C24—C25—H25A	108.9
C15—C9—H9A	110.0	C26—C25—H25A	108.9
C10—C9—H9A	110.0	C24—C25—H25B	108.9
C11—C10—C16	112.2 (3)	C26—C25—H25B	108.9
C11—C10—C9	103.2 (3)	H25A—C25—H25B	107.7
C16—C10—C9	118.9 (3)	O8—C26—C25	113.7 (3)
C11—C10—H10	107.3	O8—C26—C16	107.0 (3)
C16—C10—H10	107.3	C25—C26—C16	111.9 (3)
C9—C10—H10	107.3	O8—C26—H26	108.0
C12—C11—C10	107.3 (3)	C25—C26—H26	108.0
C12—C11—H11A	110.2	C16—C26—H26	108.0
C10—C11—H11A	110.2	O8—C27—H27A	109.5
C12—C11—H11B	110.2	O8—C27—H27B	109.5
C10—C11—H11B	110.2	H27A—C27—H27B	109.5
H11A—C11—H11B	108.5	O8—C27—H27C	109.5
O3—C12—C8	113.3 (3)	H27A—C27—H27C	109.5
O3—C12—C11	109.0 (3)	H27B—C27—H27C	109.5
C8—C12—C11	100.3 (3)	O10—C28—C23	109.6 (3)
O3—C12—C13	111.6 (3)	O10—C28—H28A	109.7
C8—C12—C13	110.2 (3)	C23—C28—H28A	109.7
C11—C12—C13	111.9 (3)	O10—C28—H28B	109.7
O5—C13—C14	107.7 (3)	C23—C28—H28B	109.7
O5—C13—C12	108.6 (3)	H28A—C28—H28B	108.2
C14—C13—C12	114.6 (3)	O10—C29—H29A	109.5
O5—C13—H13	108.6	O10—C29—H29B	109.5
C14—C13—H13	108.6	H29A—C29—H29B	109.5
C12—C13—H13	108.6	O10—C29—H29C	109.5
O4—C14—C13	107.0 (3)	H29A—C29—H29C	109.5
O4—C14—C15	112.3 (3)	H29B—C29—H29C	109.5
C13—C14—C15	117.7 (3)	N1—C30—H30A	109.5
O4—C14—H14	106.4	N1—C30—H30B	109.5
C13—C14—H14	106.4	H30A—C30—H30B	109.5
C15—C14—H14	106.4	N1—C30—H30C	109.5
O6—C15—C9	105.0 (3)	H30A—C30—H30C	109.5
O6—C15—C14	109.3 (3)	H30B—C30—H30C	109.5
C9—C15—C14	111.8 (3)	O5—C31—H31A	109.5
O6—C15—C18	107.8 (3)	O5—C31—H31B	109.5
C9—C15—C18	108.3 (3)	H31A—C31—H31B	109.5
C14—C15—C18	114.1 (3)	O5—C31—H31C	109.5
C17—C16—C26	117.4 (3)	H31A—C31—H31C	109.5
C17—C16—C21	98.4 (3)	H31B—C31—H31C	109.5
C26—C16—C21	112.9 (3)	C32—O11—H11	109.5
C17—C16—C10	106.4 (3)	O11—C32—H32A	109.5
C26—C16—C10	106.2 (3)	O11—C32—H32B	109.5
C21—C16—C10	115.6 (3)	H32A—C32—H32B	109.5
C18—C17—N1	112.9 (3)	O11—C32—H32C	109.5
C18—C17—C16	100.5 (3)	H32A—C32—H32C	109.5
N1—C17—C16	112.1 (3)	H32B—C32—H32C	109.5
C18—C17—H17	110.3		

### Hydrogen-bond geometry (Å, °)

**Table:**

*D*—H···*A*	*D*—H	H···*A*	*D*···*A*	*D*—H···*A*
O3—H3A···O5	0.82	2.11	2.612 (4)	119
O3—H3A···O11	0.82	2.22	2.923 (4)	144
O4—H4A···Cl1	0.82	2.30	3.083 (3)	161
O6—H6···Cl1	0.82	2.40	3.197 (3)	165
O9—H9···O11^i^	0.82	1.98	2.776 (4)	163
N1—H1···O8	0.90 (2)	2.11 (4)	2.808 (4)	134 (4)
N1—H1···O9	0.90 (2)	2.20 (4)	2.795 (5)	123 (4)
O11—H11···Cl1^ii^	0.82	2.30	3.084 (3)	158

Symmetry codes: (i) *x*+1, *y*, *z*; (ii) *x*−1/2, −*y*+1/2, −*z*+1.
